# Experimental Models and Translational Strategies in Neuroprotective Drug Development with Emphasis on Alzheimer’s Disease

**DOI:** 10.3390/molecules31020320

**Published:** 2026-01-16

**Authors:** Przemysław Niziński, Karolina Szalast, Anna Makuch-Kocka, Kinga Paruch-Nosek, Magdalena Ciechanowska, Tomasz Plech

**Affiliations:** 1 Department of Pharmacology, Chair of Pharmacology and Biology, Faculty of Health Sciences, Medical University of Lublin, 20-080 Lublin, Poland; przemyslaw.nizinski@umlub.edu.pl (P.N.); karolina.szalast@umlub.edu.pl (K.S.); anna.makuch-kocka@umlub.edu.pl (A.M.-K.); kinga.paruch-nosek@umlub.edu.pl (K.P.-N.); 2 Department of Integrated Medical Care, Faculty of Medicine, Collegium Medicum, The Mazovian Academy in Płock, 09-402 Płock, Poland; m.ciechanowska@mazowiecka.edu.pl

**Keywords:** neuroprotection, neurodegenerative diseases, disease-modifying therapies (DMT), in vitro and in vivo models, PROTAC

## Abstract

Neurodegenerative diseases (NDDs), including Alzheimer’s disease (AD), Parkinson’s disease (PD), amyotrophic lateral sclerosis (ALS) and frontotemporal dementia (FTD), are becoming more prevalent and still lack effective disease-modifying therapies (DMTs). However, translational efficiency remains critically low. For example, a ClinicalTrials.gov analysis of AD programs (2002–2012) estimated ~99.6% attrition, while PD programs (1999–2019) achieved an overall success rate of ~14.9%. In vitro platforms are assessed, ranging from immortalized neuronal lines and primary cultures to human-induced pluripotent stem cell (iPSC)-derived neurons/glia, neuron–glia co-cultures (including neuroinflammation paradigms), 3D spheroids, organoids, and blood–brain barrier (BBB)-on-chip systems. Complementary in vivo toxin, pharmacological, and genetic models are discussed for systems-level validation and central nervous system (CNS) exposure realism. The therapeutic synthesis focuses on AD, covering symptomatic drugs, anti-amyloid immunotherapies, tau-directed approaches, and repurposed drug classes that target metabolism, neuroinflammation, and network dysfunction. This review links experimental models to translational decision-making, focusing primarily on AD and providing a brief comparative context from other NDDs. It also covers emerging targeted protein degradation (PROTACs). Key priorities include neuroimmune/neurovascular human models, biomarker-anchored adaptive trials, mechanism-guided combination DMTs, and CNS PK/PD-driven development for brain-directed degraders.

## 1. Introduction

Interventions that preserve neuronal structure or function in the course of a degenerative process are known as neuroprotective therapy [1]. Neurodegeneration is characterized by the progressive loss of neuronal cell bodies together with their projections—axons, dendrites, and synapses—resulting in a stepwise impairment of neural function, ultimately leading to disability and death. Neurodegenerative disorders (NDDs) include those conditions in which neuronal loss is a primary feature [2]. Such diseases as Alzheimer’s disease (AD) and Parkinson’s disease (PD) are the most prevailing; however other NDDs, such as Huntington’s disease (HD), amyotrophic lateral sclerosis (ALS) or frontotemporal dementia (FTD), are also serious health concern [3,4]. Against the backdrop of global population aging, such interventions are urgently needed. Dementia, of which AD is the leading cause, already affects around 57 million people worldwide, and demographic modeling projects this figure to triple to 153 million by 2050 [5]. The economic implications are staggering: it has been estimated that the direct and indirect costs of AD and related dementias will exceed 3 trillion USD annually by mid-century, with the steepest proportional growth occurring in low- and middle-income countries [6]. PD, the fastest-growing neurodegenerative disorder, is predicted to affect about 25 million people by 2050, placing comparable pressure on health systems and caregivers [7]. In the light of the increasing prevalence of NDDs, the development of novel neuroprotective therapies is becoming one of the most important issues in contemporary medicine and pharmacology.

Current neuroprotective drug discovery involves several complementary strategies. For example, precision medicine frameworks emphasize aligning the ‘right’ target, drug, biomarker, participant, and trial design from the outset—an approach resulting in decades of failed Alzheimer’s programs [8]. Other efforts use computational screening, real-world data, and network biology to repurpose or reposition licensed agents whose safety, pharmacokinetics, and manufacturing processes are already well understood. This approach shortens development timelines and reduces investment risk [9]. A third pillar focuses on overcoming the blood–brain barrier (BBB) using novel delivery systems or drug formulations as nanocarriers, lipid-based formulations, and intranasal or subcutaneous delivery platforms that can deliver large or hydrophilic molecules to central targets [10]. A fourth pillar seeks multi-target or polypharmacological agents that can modulate intersecting pathways such as protein aggregation, neuroinflammation, and mitochondrial dysfunction [11]. Nevertheless, all of the above approaches require preliminary screening before conducting studies on animals or humans. In vitro neurobiology is dependent on a set of well-characterized cell systems that provide the necessary support for both mechanism-driven assays and high-throughput discovery. Cell lines are used in NDD drug research as scalable, inexpensive, and reproducible platforms to model disease-relevant mechanisms (e.g., β-amyloid/α-synuclein aggregation, toxin-induced neuronal loss), run cytotoxicity/phenotypic assays, and rapidly triage hits before more sophisticated yet expensive models [12]. Commonly used in vitro models in neuroprotection include, among others, SH-SY5Y human neuroblastoma cells, PC-12 rat pheochromocytoma cells, and LUHMES human mesencephalic dopaminergic neurons [13,14,15,16,17,18]. Advantages of immortalized lines are commercial availability, ease of handling, continuous proliferation, and relatively low cost (many also being human-derived, improving translational relevance). Key limitations are cancer-cell origin with genetic instability, immature/partial neuronal phenotypes, and inadequate functional recapitulation of mature neurons—even after differentiation—so results often require confirmation in more advanced models [19]. However, the translational pipeline from bench to bedside remains extremely selective. Analysis of the modern AD portfolio shows that 99.6% of compounds entering formal clinical testing fail to achieve regulatory approval [20]. Even when aggregated across all therapeutic areas, the probability that a new drug–indication pair will progress from a first-in-human study to market hovers between 8% and 14%, and success is particularly uncommon in adult-onset neurodegenerative disease [21]. Limited predictive validity of in vitro and animal models, heterogeneous patient biology, imperfect biomarkers for early target engagement, and escalating logistical and financial demands of late-phase trials are some of the contributing factors. In recent years, a number of both repositioned and novel molecules have been the focus of studies on NDDs as disease-modifying therapies (DMTs). This review paper provides an integrated, comparative analysis of in vitro and in vivo model types as complementary tools in neuroprotective drug discovery. It also emphasizes their translational relevance in light of the persistent gap between preclinical findings and clinical outcomes. Additionally, the review summarizes recent advances in DMT strategies, paying particular attention to repurposed and repositioned molecules as a promising area of research in neuroprotection in particular in AD treatment (as the far most prevalent neurodegenerative disease—more than 55 million cases in 2024) [22]. This comprehensive, up-to-date approach aims to provide a coherent framework that links experimental models with emerging pharmacological interventions. The future directions in both preclinical and clinical studies, including innovative approaches such as PROTACs, will also be incorporated.

As the literature search engine, the browsers in PubMed, Scopus, Web of Science databases and ClinicalTrials.gov register were employed. Documents published between 2000 and 2025 were only screened for eligibility. In order to qualify studies for the review, the following exclusion criteria have been applied: studies that have not been peer-reviewed, works published in languages other than English, and papers published before 2000.

## 2. In Vitro Models Used in Neuroprotection Research

In vitro systems remain the primary methodological foundation for mechanistic studies in neurodegenerative diseases, providing controlled and reproducible environments that enable systematic exploration of cellular vulnerability, oxidative stress, mitochondrial impairment, protein aggregation, synaptic failure, and neuroinflammatory signaling [23,24]. These models serve as a critical first stage in neuroprotective drug discovery, allowing researchers to evaluate cytotoxicity, pharmacodynamics, intracellular signaling and protective efficacy prior to validation in more complex in vivo systems [25]. However, the intrinsic complexity of neurodegenerative disorders—including AD, PD, ALS, and FTD—cannot be fully recapitulated in any single in vitro platform. Therefore, researchers employ a continuum of complementary model types (Table 1), each contributing specific mechanistic insights while compensating for limitations of others [26].

### 2.1. Immortalized Neuronal and Neuroblastoma-Derived Cell Lines

Immortalized neuronal cell lines remain indispensable tools in early-stage neuroprotection research, primarily due to their robustness, scalability, genetic manipulability, and suitability for high-throughput screening. However, their translational value depends strongly on how well a given line reflects disease-relevant neuronal subtypes and pathogenic mechanisms [27,28,29]. However, immortalization alters cellular physiology, leading to rapid proliferation, incomplete neuronal differentiation, and reduced ability to form stable synaptic networks, which limit their fidelity to adult human neurons [30]. A comparative summary of these immortalized models is provided in Table 1, including their neuronal subtype specificity and disease-oriented applications.

The SH-SY5Y human neuroblastoma line is the most widely used in vitro model in AD and PD research [31]. Upon differentiation (e.g., retinoic acid ± BDNF), SH-SY5Y cells acquire a post-mitotic phenotype with enhanced neurite outgrowth, increased expression of MAP2 and βIII-tubulin, and reduced proliferation. Importantly, these cells retain a mixed catecholaminergic–cholinergic phenotype, which—while sometimes viewed as a limitation—confers relevance to AD, where degeneration of basal forebrain cholinergic neurons is a central pathological feature [32]. This dual neurotransmitter identity is conceptually important when comparing models (see Table 1). SH-SY5Y cells are therefore extensively used to model Aβ-induced toxicity, tau hyperphosphorylation, oxidative stress, and mitochondrial dysfunction, as well as toxin-induced dopaminergic injury (e.g., MPP+, 6-OHDA, rotenone) [33,34,35].

PC12 cells, derived from rat pheochromocytoma, differentiate into neuron-like cells in response to nerve growth factor (NGF), activating TrkA-mediated survival signaling pathways. They are particularly useful for studying neurotrophic mechanisms, mitochondrial stress, and catecholaminergic vulnerability in PD-related paradigms [36]. Nevertheless, their non-human origin and tumor-derived background limit direct translational relevance (Table 1).

LUHMES cells represent a more advanced human dopaminergic in vitro system. Following differentiation, LUHMES cells acquire a homogeneous, post-mitotic phenotype with robust expression of TH, DAT, and VMAT2, functional electrophysiological properties, and high sensitivity to mitochondrial toxins and α-synuclein overexpression. These features make LUHMES cells particularly valuable for mechanistic PD studies and neuroprotective screening. Their main limitations include technical complexity and limited suitability for long-term exposure paradigms [37].

In the context of ALS, NSC34 cells—hybrid cells generated by fusion of mouse motor neurons with neuroblastoma cells—constitute one of the most commonly used immortalized motor neuron-like models. NSC34 cells express motor neuron markers (e.g., ChAT, HB9) and are widely employed to study SOD1-, TDP-43-, and C9orf72-related toxicity, excitotoxicity, mitochondrial dysfunction, and proteostasis impairment. Although they lack full electrophysiological maturity, NSC34 cells provide a cost-effective and disease-relevant platform for early ALS-focused neuroprotection studies [38,39,40].

Other immortalized lines (Neuro-2a, N27, MN9D) remain useful for specific mechanistic questions but are generally limited to early discovery stages due to incomplete neuronal differentiation and restricted disease specificity [19,41]. Table 1 summarizes in vitro neurodegeneration models with respect to their cellular origin, neuronal subtype relevance, and disease-oriented applications, enabling comparison across different neurodegenerative disorders.

**Table 1:** Summary of experimental in vitro platforms used to model neurodegenerative mechanisms.

Model Type	Representative Examples	Key Properties	Main Applications	Ref.
**Immortalized neuronal/neuroblastoma-derived lines**	SH-SY5Y, PC12, LUHMES, Neuro-2a, N27, MN9D, NSC34	Robust proliferation; easy genetic manipulation; line-specific neuronal subtype bias; incomplete neuronal maturity	High-throughput neurotoxicity screening, mitochondrial and oxidative stress assays, mechanistic studies across AD-, PD- and ALS-related paradigms	[27,30,32,33,36,37,39,40,42,43,44,45]
**Primary neuronal cultures**	Rodent cortical, hippocampal, midbrain neurons	Native electrophysiology; authentic neurotransmitter identity; natural glial environment	Synaptic physiology; toxin-induced degeneration; validation of neuroprotective candidates	[46,47,48,49,50,51,52]
**iPSC-derived** **neuronal and glial cells**	iPSC-derived cortical, dopaminergic, cholinergic, motor neurons; iPSC astrocytes, microglia	Patient-specific genetics; disease-relevant phenotypes in the presence of pathogenic mutations or defined stressors; human lineage specificity	Modeling familial/sporadic AD, PD, ALS, FTD; testing genotype-specific therapies	[23,39,40,44,45,53,54,55,56]
**Neuron–glia** **co-cultures**	Neuron–astrocyte, neuron–microglia, neuron–oligodendrocyte	Multicellular interactions; neuroinflammation modeling	Studying glial contribution to neurodegeneration; inflammatory neurotoxicity; metabolic coupling	[57,58,59,60,61,62,63,64,65,66,67]
**Microglia cultures**	Primary microglia, iPSC-microglia	TLR4 signaling; cytokine response; phagocytosis	Modeling neuroinflammation, screening anti-inflammatory compounds	[56,65,66,68,69,70]
**Three-dimensional spheroids/scaffold-based constructs**	Three-dimensional neuronal spheroids, ECM-supported constructs	Three-dimensional architecture; enhanced cell–cell contact; early aggregation biology	Modeling Aβ, tau, α-syn aggregation; testing neuroprotective agents in tissue-like setups	[50,71,72,73,74]
**Microfluidic platforms**	Axon–soma chips, neuron–glia-on-chip, AD-on-chip, PD-on-chip	Compartmentalization; controlled gradients; directional propagation	Studying axonal degeneration, synaptic vulnerability, propagation of Aβ/tau/α-syn	[75,76,77,78,79,80]
**BBB-on-chip systems**	Endothelial–astrocyte–pericyte chips	Barrier function; dynamic flow; transporter activity	Drug penetration assays; modeling BBB breakdown in AD and PD	[81,82,83,84,85]
**Brain organoids**	Cortical, midbrain, hippocampal, spinal, multi-region assembloids	Highest complexity; layered cytoarchitecture; self-organizing networks	Modeling AD/PD/ALS/FTD pathology, testing disease-modifying therapies	[26,86,87,88,89,90,91,92,93,94,95,96,97,98,99,100]

### 2.2. Primary Neuronal Cultures

Primary neuronal cultures derived from rodent cortex, hippocampus, or ventral mesencephalon provide substantially higher physiological relevance than immortalized cell lines due to their native electrophysiology, neurotransmitter identity, synaptic organization, and metabolic profile [46,47,48]. These cultures naturally include multiple neuronal subtypes and glial cells, creating a microenvironment that more accurately reflects in vivo interactions. Midbrain primary cultures are especially valuable for modeling PD, as they contain endogenous dopaminergic neurons with authentic DAT and TH expression and recapitulate their characteristic sensitivity to mitochondrial toxins, oxidative stress, and excitotoxicity [49,50]. Their position within the in vitro hierarchy relative to immortalized lines and iPSC models is presented in Table 1.

**Figure 1:**
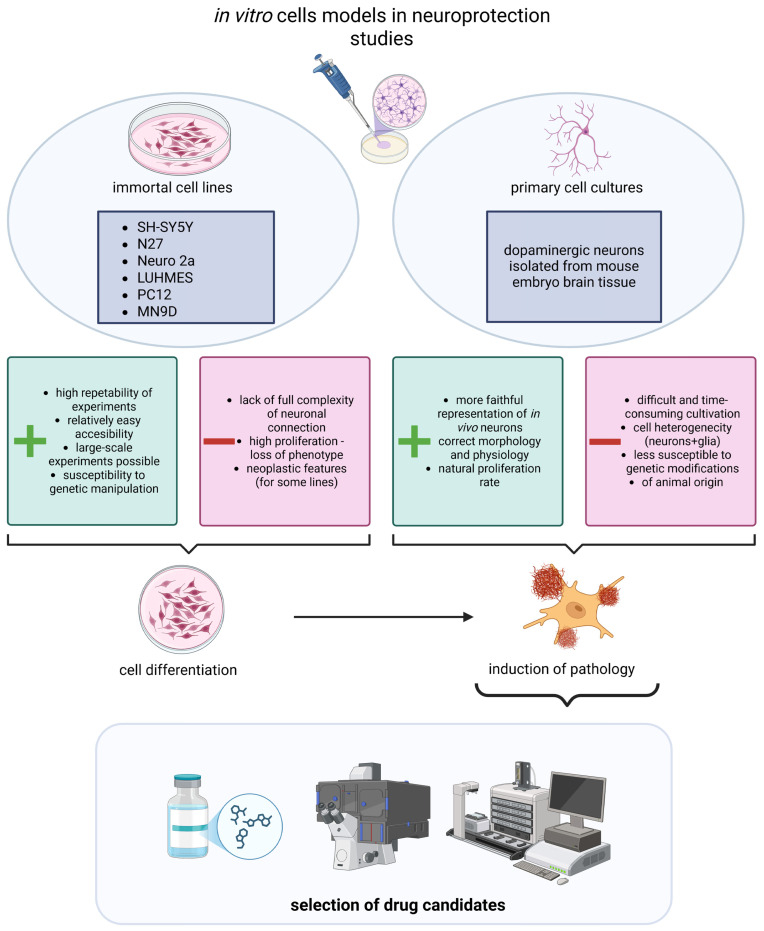
Comparison of immortalized neuronal cell lines and primary neuronal cultures used in neuroprotection studies. The figure summarizes key advantages and limitations of immortalized cell models versus primary neurons, highlighting their differing suitability for neuroprotection studies. Created in BioRender^®^.

However, despite their physiological fidelity, primary neurons face substantial practical limitations: they are technically demanding to prepare, generate low yields, exhibit high batch-to-batch variability, and have limited viability in long-term assays [51]. Furthermore, interspecies differences—particularly between rodent and human neurons—introduce translational challenges that must be considered when interpreting neuroprotective effects [52] (Figure 1). These characteristics position primary cultures as a bridge between immortalized systems and stem cell-derived human neurons, as outlined in Table 1.

### 2.3. Induced Pluripotent Stem Cell (iPSC)-Derived Neuronal Models

Induced pluripotent stem cell (iPSC)-derived neuronal models represent a major advance in neurodegeneration research due to their human origin and capacity to preserve patient-specific genetic backgrounds (Table 1). Importantly, iPSC-derived neurons do not intrinsically display disease phenotypes [86,87]. Disease-relevant features emerge only when pathogenic mutations are present (e.g., *APP*, *PSEN1/2*, *SNCA*, *C9orf72*, *TDP-43*, *FUS*) or when defined pathological stressors are applied (e.g., oxidative stress, proteotoxic challenge, inflammatory cues) [53,54,101].

iPSC-derived cortical neurons carrying familial AD mutations reproduce key pathological hallmarks, including increased Aβ42/40 ratios, tau hyperphosphorylation, synaptic loss, and mitochondrial impairment. Similarly, APOE4-expressing iPSC neurons and astrocytes exhibit lipid dysregulation, impaired Aβ clearance, and heightened vulnerability to oxidative stress—phenotypes absent in mutation-free controls [52,101].

iPSC-derived dopaminergic neurons modeling PD-associated mutations (*SNCA*, *LRRK2*, *PINK1*, *Parkin*, *GBA*) recapitulate α-synuclein aggregation, lysosomal dysfunction, impaired mitophagy, and mitochondrial vulnerability. In ALS and FTD research, iPSC-derived motor neurons and cortical neurons carrying *SOD1*, *TDP-43*, *FUS*, or *C9orf72* mutations reproduce hallmark phenotypes such as TDP-43 mislocalization, DPR accumulation, axonal transport deficits, and early synaptic dysfunction [102].

Thus, iPSC-based systems function as genetically and mechanistically defined disease models, rather than generic neuronal surrogates, and are best interpreted within this context.

### 2.4. Neuron–Glia Co-Culture and Microglia-Based Models

Growing evidence indicates that neurodegeneration is not solely a neuronal process but results from pathological interactions between neurons, astrocytes, microglia, and oligodendrocytes. Co-culture systems therefore represent a crucial intermediate between classical monolayers and 3D models, as they preserve multicellular communication necessary for modeling synaptic dysfunction, neuroinflammation, oxidative stress, and metabolic coupling [57,58,59]. Their functional relevance within the broader landscape of in vitro platforms is outlined in Table 1.

#### 2.4.1. Astrocyte–Neuron Co-Cultures

Astrocytes play a central role in glutamate homeostasis, metabolic support, antioxidant defense, and synaptic modulation. In AD models, co-cultures reveal how Aβ-exposed astrocytes impair synaptic plasticity, alter calcium signaling, and contribute to neuronal death [60,61]. In PD, astrocytes derived from *LRRK2* or *GBA* mutation carriers demonstrate defective lysosomal activity and decrease neuroprotective capacity, amplifying α-synuclein toxicity [62,63]. Astrocytes derived from ALS patients exhibit reduced glutamate uptake due to EAAT2 downregulation, causing excitotoxic stress and selective motor neuron vulnerability [64].

#### 2.4.2. Microglia–Neuron Co-Cultures

Microglia are primary mediators of neuroinflammation, and microglia–neuron interactions strongly influence the course of PD, AD, ALS, and FTD. In vitro activation of microglia using lipopolysaccharide (LPS)—one of the most widely used methods for inducing neuroinflammation—leads to release of TNF-α, IL-1β, nitric oxide, and ROS, which induce neuronal dysfunction and apoptosis [65,68]. LPS-activated microglia also exacerbate tau phosphorylation, stimulate Aβ production, promote α-synuclein aggregation, and drive TDP-43 mislocalization, providing pathophysiologically relevant mechanisms across neurodegenerative diseases [69,103].

#### 2.4.3. Microglia Models

Microglia-derived cultures—either from primary rodent tissue or iPSC-derived microglia—enable the modeling of innate immune activation, phagocytosis deficits, cytokine release, and inflammasome signaling. They are essential for investigating neuroinflammation-driven neurotoxicity and the protective role of anti-inflammatory agents [70].

#### 2.4.4. LPS-Induced Neuroinflammation

LPS is one of the most widely used agents for inducing neuroinflammation in vitro. LPS stimulates microglial activation via Toll-like receptor 4 (TLR4), increasing release of TNF-α, IL-1β, IL-6, NO, and ROS, resulting in secondary neuronal injury. LPS-based models are crucial for evaluating anti-inflammatory and neuroprotective compounds and are now considered essential in modern neurodegeneration research [66].

### 2.5. Three-Dimensional Neuronal Spheroids and Scaffold-Based Models

Three-dimensional neuronal spheroids, generated from primary neurons, immortalized lines, or iPSC-derived cells, reproduce essential aspects of tissue architecture absent from 2D monolayers, including gradients of oxygen, nutrients, and metabolites; enhanced cell–cell communication; and early extracellular matrix formation [88,89,104]. These properties promote more realistic modeling of protein aggregation, including Aβ plaques, tau tangles, and α-synuclein inclusions. Their comparative complexity relative to monolayers and organoids is mapped in Table 1.

In AD spheroids, Aβ accumulation emerges spontaneously due to limited diffusion, and tau pathology develops in response to prolonged neuronal stress [71,105]. In PD spheroids, α-synuclein aggregation, mitochondrial deficits, and dopaminergic degeneration appear more prominently than in 2D cultures [106]. ALS spheroids reveal early neuromuscular junction deficits and TDP-43 mislocalization [107]. Three-dimensional neuronal spheroids and scaffold-based constructs provide improved cellular architecture, ECM-like interactions, and gradients of oxygen and nutrients that more closely mimic in vivo environments. While spheroids lack full spatial heterogeneity of organoids, they provide a highly scalable, cost-effective, and translationally relevant mid-complexity system suitable for neuroprotective compound testing [72,73,74].

### 2.6. Microfluidic Platforms and Brain-on-a-Chip Systems

Microfluidic devices enable compartmentalized cultures that precisely control fluid dynamics, axonal growth, synaptic connectivity, and directional signaling [75,76,77]. These systems mimic neuronal circuitry more accurately than monolayers and allow researchers to study axonal degeneration, synaptic vulnerability, and neuron–glia communication under physiologically relevant spatial constraints. In PD-on-chip models, microfluidic separation of soma and axons allows specific induction of mitochondrial stress or α-synuclein propagation along axonal compartments [78,108,109]. In AD-on-chip systems, microfluidic gradients induce directional Aβ diffusion, enabling analysis of synaptic impairment and tau spreading. Microfluidic devices are also widely used to model neuroinflammation: LPS-activated microglia placed in separate chambers influence neuronal compartments via cytokine gradients, providing a physiologically realistic neuroimmune interface [67].

These features place microfluidic platforms at an intermediate-to-high level of complexity within the in vitro modeling spectrum, as summarized in Table 1.

### 2.7. Blood–Brain Barrier (BBB)-on-Chip Systems

BBB-on-chip platforms integrate endothelial cells, astrocytes, and pericytes within microfluidic devices to reproduce barrier integrity, transendothelial resistance, transporter activity, and molecular permeability [81,82]. These systems are essential for neuroprotection research, as many therapeutic candidates fail due to inadequate BBB penetration. In AD, BBB dysfunction is increasingly recognized as a major early pathogenic mechanism, associated with APOE4 status, pericyte degeneration, reduced Aβ clearance, and altered glymphatic drainage [83]. BBB-on-chip systems allow mechanistic studies of these processes and provide a translational platform for evaluating CNS delivery of drug candidates [84,85].

Their role in bridging cellular neurobiology with pharmacokinetics and drug delivery considerations is reflected in the comparative overview presented in Table 1.

### 2.8. Brain Organoids and Region-Specific Neural Organoids

Brain organoids represent one of the most advanced modeling strategies currently available, recapitulating aspects of human brain cytoarchitecture, cellular diversity, layered organization, and developmental trajectories far beyond the capabilities of 2D cultures or 3D spheroids [90,91]. Generated from human pluripotent stem cells (hESCs or iPSCs), organoids differentiate into heterogeneous populations of neurons, astrocytes, radial glia, and—in more mature constructs—oligodendrocyte precursors and microglia-like cells [91,92]. While they do not fully reproduce vascularization, long-range connectivity, or adult maturation, organoids provide an exceptionally powerful human-based platform for investigating neurodevelopmental and neurodegenerative mechanisms [94].

Alzheimer’s organoids recapitulate extracellular Aβ plaque deposition, intracellular tau hyperphosphorylation, synaptic disruption, and neuronal network dysregulation [92,95]. iPSC-derived organoids carrying familial AD mutations (*APP*, *PSEN1*, *PSEN2*) exhibit accelerated Aβ42/40 ratios, enhanced plaque formation, increased tau misfolding, and mitochondrial dysfunction, mirroring early pathogenic cascades observed in human AD brains [91]. APOE4 -dependent phenotypes—including lipid dysregulation, impaired Aβ clearance, and increased neuroinflammation—are consistently observed in AD organoids, providing a translationally relevant platform for testing neuroprotective compounds, anti-Aβ antibodies, anti-tau agents, or modulators of lipid metabolism [93].

Midbrain organoids containing dopaminergic neurons model PD pathology with higher fidelity than monolayers [96]. They develop neuromelanin-like granules, α-synuclein accumulation, mitochondrial impairment, and dopaminergic cell loss, particularly in organoids generated from *LRRK2*, *SNCA*, *Parkin*, or *PINK1* mutation carriers [97]. These constructs are increasingly used to study mitochondrial quality control, mitophagy, and α-synuclein propagation—processes that are insufficiently represented in SH-SY5Y or LUHMES cultures [98]. Exposure to PD toxins (MPP+, rotenone) reproduces dopaminergic vulnerability within a 3D microenvironment, enabling evaluation of neuroprotective strategies targeting mitochondrial stabilization, oxidative stress reduction, or α-synuclein clearance.

Cortical organoids derived from patients with *C9orf72*, *TDP-43*, *FUS*, or *MAPT* mutations exhibit hallmark pathological features including TDP-43 mislocalization, DPR accumulation, defective autophagy, disrupted RNA metabolism, and early synaptic degeneration [99]. Organoid-based motor neuron systems reveal impaired axonal transport, defective neuromuscular junction formation, and increased susceptibility to excitotoxic stress. These properties make organoids uniquely suited for studying ALS–FTD spectrum disorders, where cortical vulnerability and frontotemporal circuit degeneration cannot be reproduced in traditional 2D cultures [93].

Despite their strengths, organoids exhibit limited reproducibility, inter-batch variability, and immature neuronal phenotypes and lack vasculature or microglial integration unless artificially introduced. Their prolonged culture requirements (weeks to months) also limit throughput [90]. Nevertheless, organoids currently represent the most human-relevant in vitro platform available, offering the closest approximation of human neurodegenerative processes and enabling mechanistic and therapeutic studies that were previously feasible only in animal models [91]. Their position within the experimental hierarchy is illustrated in Table 1, emphasizing their complementary role alongside other in vitro systems.

### 2.9. Comparative Context and Integration of In Vitro Approaches

No single in vitro model is sufficient to capture the multifactorial nature of neurodegenerative diseases, which involve complex interactions between genetic risk factors, environmental stressors, mitochondrial dysfunction, proteostasis imbalance, neuroinflammation, synaptic failure, and circuit-level disintegration [79]. Immortalized lines provide mechanistic clarity for early high-throughput screening; primary cultures add physiological relevance; co-cultures incorporate glial contributions; iPSC-derived neurons enable patient-specific modeling; microfluidic systems simulate spatial organization; and organoids reflect multicellular complexity and partially mimic tissue architecture [80].

Importantly, certain models align more strongly with particular diseases. PD studies benefit from dopaminergic LUHMES cells, midbrain primary cultures, microfluidic axonal chips, and midbrain organoids [100]. AD research requires Aβ- and tau-specific systems such as cortical iPSC neurons, APOE4-dependent models, and AD organoids with amyloid deposition. ALS and FTD require models capable of reproducing TDP-43 pathology, DPR accumulation, and axonal degeneration, achievable in iPSC motor neurons, corticospinal circuits, and region-specific organoids [110,111]. This disease-specific mapping is summarized in Table 2, while the alignment between mechanistic domains and model choice is further detailed in Table 3.

In vitro models provide an indispensable spectrum of tools for dissecting cellular mechanisms of neurodegeneration and for screening neuroprotective strategies. Their modularity allows targeted investigation of mitochondrial dysfunction, oxidative stress, proteostasis imbalance, neuroinflammation, and synaptic failure across multiple neurodegenerative disorders [79]. However, in vitro systems inherently lack the vascular, immune, and circuit-level interactions characteristic of intact nervous systems and cannot reproduce behavioral or systemic responses to pathology [56]. Consequently, findings obtained in vitro require validation in animal models capable of reproducing disease progression, neuroimmune responses, network-level degeneration, and pharmacokinetic/pharmacodynamic interactions [114]. This conceptual continuum—from molecular in vitro systems to whole-organism in vivo models—forms the basis for translational neuroprotection research. The following chapter addresses in vivo approaches, highlighting toxin-based, genetic, and advanced animal models used to study PD, AD, ALS, and FTD, and illustrating how they complement the in vitro systems described above [115]. A comparative summary of disease-relevant mechanism across models is provided in Table 3.

**Table 3:** Mapping of core neurodegenerative mechanisms to appropriate in vitro models.

Mechanistic Domain	Optimal In Vitro Systems	Examples of Readouts	Primary Diseases Modeled	Ref.
**Mitochondrial dysfunction**	SH-SY5Y, LUHMES, iPSC-derived dopaminergic/motor neurons, 3D spheroids	MMP, OCR, ATP production, ROS, mitophagy flux	PD, AD, ALS	[24,33,40,43,45,50,109,116]
**Protein aggregation (Aβ** **, tau, α** **-syn, TDP-43, DPRs)**	iPSC neurons, 3D spheroids, AD/PD organoids, co-cultures	Aβ42/40, tau-P, α-syn fibrils, TDP-43 loss of function	AD, PD, ALS, FTD	[23,34,42,95,99,100,104,105,106,107]
**Neuroinflammation**	Microglia monoculture, neuron–microglia co-cultures, LPS models, organoids with microglia	TNF-α, IL-1β, NO, ROS, inflammasome activation	AD, PD, ALS, FTD	[62,63,64,65,66,67,68,70,93,113]
**Synaptic dysfunction**	iPSC cortical neurons, primary hippocampal neurons, organoids	Synaptic density, PSD-95, synaptophysin, electrophysiology	AD, FTD	[34,46,48,92,103,105]
**Axonal degeneration**	Microfluidic axon-on-chip, iPSC motor neurons, 3D motor neuron constructs	Axon length, transport dynamics	ALS, PD	[44,45,49,75,78,80]
**Excitotoxicity**	Primary neurons, iPSC cortical/motor neurons, co-cultures	Ca^2+^ imaging, glutamate toxicity	ALS, AD	[60,64,66]
**Lysosomal/autophagy deficits**	iPSC dopaminergic neurons, LUHMES, PD organoids	LC3-II, p62, lysosomal pH	PD, AD	[40,63,99,109,117]
**BBB dysfunction**	BBB-on-chip models	TEER, permeability, transporter function	AD, PD	[81,82,83,84,85]

## 3. In Vivo Models Used in Neuroprotection Research

**Figure 2:**
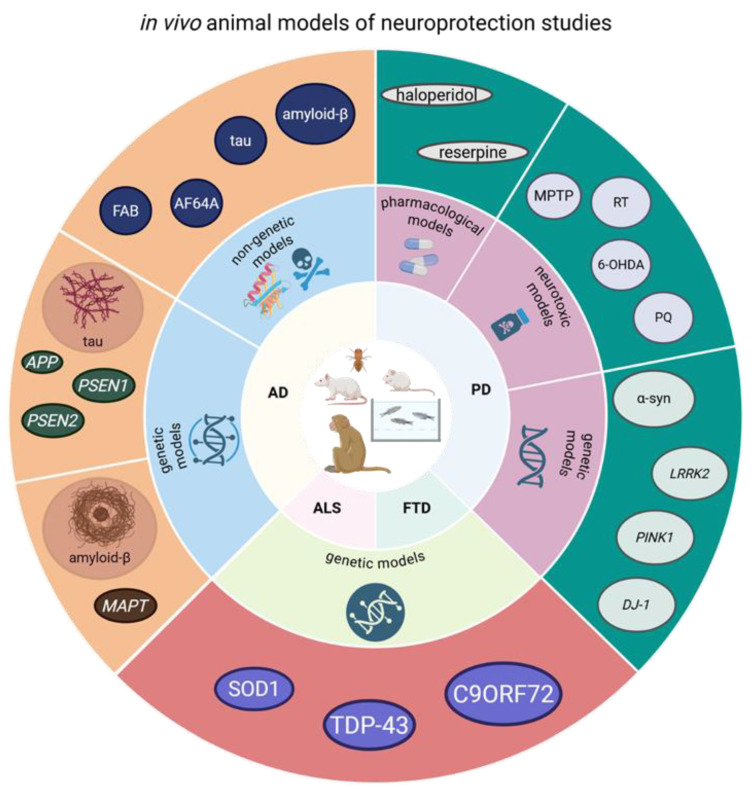
Genetic and non-genetic factors associated with major neurodegenerative diseases. The figure illustrates the division of pathogenic contributors into genetic and non-genetic categories across AD, PD, ALS, and FTD. Created in BioRender^®^.

In vivo experimental systems remain a critical component of preclinical neuroprotection research because they provide levels of biological complexity, systemic interactions, and long-range neuronal connectivity that cannot be reproduced in vitro [118,119,120]. While advanced in vitro models—such as organoids, iPSC-derived neuronal systems, microfluidic platforms, and 3D co-cultures—offer increasingly human-relevant environments, they lack the integrated neurovascular, hormonal, metabolic, inflammatory, and behavioral dimensions required to fully recapitulate neurodegenerative disease progression [121,122,123,124]. Animal models enable the evaluation of pharmacokinetics, drug metabolism, blood–brain barrier (BBB) penetration, and behavioral phenotypes, all of which are essential for translating mechanistic insights into clinically relevant neuroprotective strategies [125]. However, neurodegenerative disorders such as PD, AD, ALS, and FTD are heterogeneous at the molecular, cellular, and systems levels, and no single model fully encompasses their multifactorial pathology [126,127]. Historically, in vivo models were categorized strictly by disease (e.g., “PD models”, “AD models”), but this approach often obscured the underlying pathophysiological processes they represent and contributed to poor translational performance [128,129]. Overall relationships between major categories of in vivo models and their relevance to neurodegenerative disease mechanisms are summarized in Figure 2.

### 3.1. Toxin-Based In Vivo Models of Neurodegeneration

Toxin-based animal models have long served as foundational tools for investigating mechanistic pathways of neurodegeneration and evaluating the neuroprotective potential of novel therapeutic agents [130,131,132]. Their translational relevance stems from the fact that many neurotoxins produce highly reproducible and region-specific patterns of neuronal vulnerability, particularly in systems implicated in PD, AD, ALS, and FTD. Although these models do not fully reproduce the multifactorial pathophysiology of human disorders, they remain indispensable for studying oxidative stress, mitochondrial damage, excitotoxicity, protein aggregation, synaptic dysfunction, and glial activation—mechanisms that represent conserved pathogenic hubs across multiple neurodegenerative conditions [133,134] (Table 4).

One of the earliest and most influential toxin-based models is reserpine, a vesicular monoamine transporter-2 (VMAT2) inhibitor that leads to depletion of dopamine, norepinephrine, and serotonin stores. Systemic administration of reserpine induces profound akinesia, rigidity, ptosis, and hypothermia in rodents, mimicking key motor symptoms of PD [135,136]. This phenotype is accompanied by elevated glutamate release in basal ganglia nuclei and hyperactivity of the subthalamic nucleus, paralleling pathophysiological hallmarks of PD circuitry. While reserpine does not cause degeneration of dopaminergic neurons and therefore lacks face validity for progressive PD, its predictable induction of motor deficits makes it valuable for screening symptomatic therapies, particularly dopaminergic agents [137].

Another frequently used pharmacological model is haloperidol, a dopamine D2 receptor antagonist that induces catalepsy, bradykinesia, and muscle rigidity in rodents and primates. Haloperidol administration disrupts basal ganglia output through post-synaptic receptor blockade rather than neuronal loss, allowing investigators to differentiate between dopamine-dependent motor impairments and degenerative nigrostriatal damage [138,139]. As a model, haloperidol is particularly useful for examining extrapyramidal side effects of antipsychotic medications and for studying compensatory responses to impaired dopaminergic signaling. More pathologically relevant toxin-based models induce structural neuronal loss similar to those observed in human PD [140].

The best-characterized example is 1-methyl-4-phenyl-1,2,3,6-tetrahydropyridine (MPTP), which readily crosses the BBB and is metabolized by monoamine oxidase-B into the active metabolite MPP+. MPP+ selectively enters dopaminergic neurons via the dopamine transporter (DAT), where it disrupts mitochondrial complex I, causes ATP depletion, and generates reactive oxygen species [141]. These events trigger apoptotic and necrotic pathways, microglial activation, and progressive dopaminergic neurodegeneration [142,143]. MPTP models—particularly in mice and non-human primates—remain among the most translationally informative tools for studying PD mechanisms and evaluating neuroprotective agents targeting mitochondrial preservation, antioxidant defense, and inflammatory modulation.

A second widely applied toxin is 6-OHDA, a catecholaminergic neurotoxin that produces profound, often unilateral, degeneration of dopaminergic projections when injected directly into the substantia nigra or medial forebrain bundle [144]. Because 6-OHDA does not cross the BBB, its use requires stereotactic injection, allowing precise control over the extent and anatomical localization of lesions. This model produces robust motor deficits, including rotational behavior, gait impairment, and postural instability [145]. Although 6-OHDA does not induce intracellular α-synuclein aggregation or Lewy body formation—key pathological hallmarks of human PD—it remains central to preclinical research due to its reproducibility, rapid onset, and suitability for evaluating restorative strategies such as cell replacement, neurotrophic factors, and gene therapies [146].

The environmental pesticides of rotenone and paraquat constitute additional mitochondrial toxins with high relevance to PD and potentially other neurodegenerative disorders. Rotenone, a complex I inhibitor with high lipophilicity, crosses the blood–brain barrier and induces dopaminergic degeneration, oxidative stress, lipid peroxidation, and cytoplasmic α-synuclein inclusions resembling Lewy bodies [147]. Paraquat, structurally similar to MPP+, promotes redox cycling, ROS overproduction, and α-synuclein aggregation, although its high systemic toxicity and variability in central nervous system penetration pose challenges for reproducibility [148,149]. Across these toxin-based models, a unifying feature is their ability to generate oxidative stress, mitochondrial dysfunction, and cytoskeletal alterations—mechanisms central to multiple neurodegenerative diseases [150]. Although they lack the slow, progressive nature of human disorders and do not reproduce complex genetic–environmental interactions, their mechanistic specificity and robust phenotype generation make them invaluable for dissecting early pathogenic cascades and evaluating candidate neuroprotective drugs [151].

**Table 4:** Toxin-based in vivo models of neurodegeneration and their mechanistic relevance.

Model	Primary Mechanism	Main Phenotype	Relevance to Human Disease	Ref.
**Reserpine**	VMAT2 inhibition; monoamine depletion	Akinesia, rigidity, hypothermia	Symptom mimicry in PD; no neurodegeneration	[135,136,137]
**Haloperidol**	D2 receptor blockade	Catalepsy, bradykinesia	Extrapyramidal symptoms; post-synaptic dysfunction	[138,139,140]
**MPTP/MPP+**	Mitochondrial complex I inhibition; oxidative stress	Loss of SNpc dopaminergic neurons, microgliosis	Strong mechanistic resemblance to PD	[130,131,134,141,143]
**6-OHDA**	DAT-mediated catecholaminergic toxicity; oxidative damage	Unilateral degeneration, robust motor deficits	Lesion-based PD model; high reproducibility	[133,134,145,146]
**Rotenone**	Mitochondrial inhibition; lipid peroxidation	α-Synuclein inclusions, dopaminergic loss	Environmental PD risk modeling	[131,132,147,148]
**Paraquat**	Redox cycling; ROS generation; α-syn aggregation	Oxidative stress, neuronal death	PD-related oxidative pathology; high systemic toxicity	[148,149,150]

### 3.2. Genetic In Vivo Models of Neurodegeneration

Genetic models provide essential insight into how inherited pathogenic variants contribute to the onset and progression of neurodegenerative disorders. Unlike toxin-based models, which induce rapid, often acute neuronal injury, genetic models can reproduce the chronic and progressive nature of human disease, enabling investigators to study molecular cascades that unfold gradually over months—more closely reflecting human pathophysiology [152,153,154]. Their value lies particularly in modeling protein misfolding, aggregation, synaptic and axonal dysfunction, mitochondrial impairment, and neuroimmune activation, which are shared with mechanistic drivers across AD, PD, ALS, and FTD [155,156,157].

Among the best-characterized genetic systems are models overexpressing or mutating *α-synuclein*, the principal component of Lewy bodies. Transgenic mice expressing human α-synuclein variants (A53T, A30P, E46K) develop progressive motor deficits, synaptic dysfunction, and intracellular aggregates reminiscent of PD pathology [158,159,160]. Viral vector-based *α-synuclein* overexpression induces robust nigrostriatal degeneration and mimics prion-like propagation of pathological aggregates, offering a powerful platform for studying neuronal–glial interactions and network-level spread [161]. Mutations in *LRRK2*, the most common cause of familial PD, have also been modeled extensively [162,163]. LRRK2 G2019S and R1441C/G knock-in mice show alterations in synaptic plasticity, dopamine release, and axonal structure, although consistent dopaminergic neurodegeneration is often absent, highlighting the partial penetrance of LRRK2 pathology in humans [164,165]. These models have proven valuable for dissecting kinase-dependent mechanisms, testing LRRK2 inhibitors, and exploring interactions with inflammatory pathways [166]. Additional PD-related genes include *Parkin* and *PINK1*, both critical regulators of mitochondrial quality control. Loss-of-function models demonstrate impaired mitophagy, synaptic dysregulation, vulnerability toxin-induced neurodegeneration, and age-dependent motor deficits [167,168]. Although these models do not always produce overt neuronal loss, they capture upstream mechanisms relevant for early PD and for evaluating mitophagy-enhancing therapies [169,170].

AD genetic models primarily focus on mutations in *APP*, *PSEN1*, and *PSEN2*, which accelerate amyloid β (Aβ) production and aggregation. Transgenic lines such as APP/PS1, 5xFAD, and Tg2576 develop early and robust amyloid plaque deposition, gliosis, synaptic degeneration, and cognitive impairment. These models have been essential for elucidating amyloid-driven toxicity, microglial reactivity, neurovascular disruption, and synaptic failure [171,172,173]. More recently, APOE4 knock-in mice have gained attention due to the allele’s strong association with sporadic AD. *APOE4* expression promotes Aβ accumulation, BBB breakdown, neuroinflammation, lipid dysregulation, and impaired neuronal resilience—closely mirroring human biomarker and imaging findings [39,174]. These models also respond differently to therapeutic interventions, enabling genotype-stratified drug evaluation [175]. Models overexpressing mutant *tau*, including P301S and P301L lines, reproduce neurofibrillary tangle formation, axonal degeneration and neuronal loss, providing a platform for testing anti-tau therapies and studying mechanisms of cytoskeletal destabilization [176,177]. Combined models that integrate *APP*, *PSEN,* and *tau* mutations accelerate neurodegeneration and offer improved construct validity by capturing more advanced stages of AD pathology [178].

Mutations in *SOD1*, particularly SOD1^G93A, remain the most widely used ALS models. These mice exhibit progressive motor neuron degeneration, axonal dieback, neuromuscular junction loss, microgliosis, and paralysis, closely paralleling human ALS progression [179]. Beyond SOD1-based systems, contemporary ALS and FTD research increasingly relies on models targeting RNA-binding protein pathology. TDP-43 transgenic and knock-in mouse models reproduce hallmark disease features including nuclear clearance and cytoplasmic aggregation of TDP-43, synaptic dysfunction, axonal degeneration, neuroinflammatory activation, and progressive motor or cognitive deficits, depending on the affected neural circuits. Although the extent of overt motor neuron loss varies across models, these systems capture core ALS/FTD proteinopathy and are widely used for mechanistic and therapeutic studies. FUS-based models further expand the ALS/FTD spectrum by recapitulating RNA dysmetabolism, impaired DNA damage response, stress granule pathology and nucleocytoplasmic transport defects. Mutant FUS expression results in synaptic dysfunction and neurodegeneration, providing insight into convergent pathways shared across ALS and FTD [180,181,182,183]. *C9orf72* repeat expansion models reproduce dipeptide repeat protein accumulation, RNA foci formation, innate immune activation, and behavioral abnormalities. While neuronal loss is often modest, these models closely mirror early-stage ALS/FTD pathology and are highly relevant for therapeutic strategies targeting RNA toxicity, proteostasis and neuroimmune pathways [184,185,186]. Animal models of frontotemporal dementia (FTD) primarily reflect the molecular heterogeneity of the disease and are commonly grouped into tauopathy-driven and RNA/proteinopathy-based systems. Transgenic mice expressing mutant tau variants (e.g., P301S and P301L) develop progressive frontotemporal neurodegeneration, synaptic loss, gliosis, and behavioral abnormalities resembling behavioral variant FTD, making them valuable platforms for studying tau-mediated neurotoxicity and for testing anti-tau therapeutic strategies. Complementary FTD models are based on TDP-43 and C9orf72 pathology, which capture non-tau FTD subtypes. These systems reproduce cortical vulnerability, synaptic dysfunction, neuroinflammatory activation and cognitive–behavioral phenotypes rather than pure motor deficits, aligning closely with the clinical presentation of FTD and reinforcing the conceptual overlap between ALS and FTD within a disease spectrum [161,176,177].

Across all genetic models, a central theme emerges: while no single model captures the full spectrum of human neurodegeneration, together they provide a mosaic of mechanistic insights essential for understanding disease progression and for evaluating disease-modifying therapies (Table 5).

### 3.3. Integrative and Emerging In Vivo Models of Neurodegeneration

**Figure 3:**
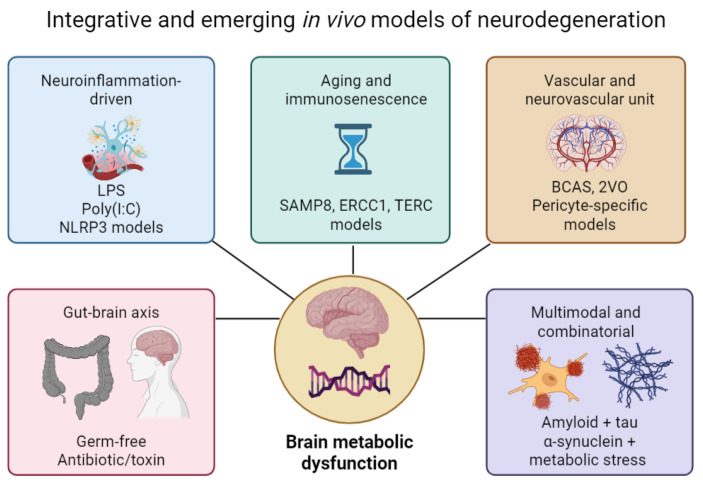
Integrative in vivo models of neurodegeneration. The figure illustrates major experimental in vivo paradigms that model key pathogenic drivers implicated in AD, PD, ALS, and FTD. Neuroinflammation-driven models, aging and immunosenescence models, vascular and neurovascular-unit models, gut–brain axis perturbations, and multimodal combinatorial paradigms converge mechanistically on brain metabolic dysfunction, a central vulnerability hub in neurodegenerative disease. Created in BioRender^®^.

As neurodegenerative diseases arise from multifactorial interactions among genetic, environmental, metabolic, immune, and age-related factors, single-mechanism in vivo models often fail to capture the full complexity of human pathology. To address this, a new generation of integrative and multimodal models has emerged, providing deeper mechanistic resolution and improved translational validity [68]. These relationships and their convergence across disease-relevant domains are illustrated in Figure 3. These systems bridge the gap between traditional models and human clinical biology by incorporating neuroinflammation, metabolic dysfunction, vascular compromise, microbiome–brain interactions, and the biology of aging—mechanisms strongly implicated in AD, PD, ALS, and FTD [189,190].

#### 3.3.1. Neuroinflammation-Driven Models

Systemic or central administration of inflammatory agents, most notably LPS, produces robust activation of microglia, cytokine release, disruption of synaptic homeostasis, and progressive neuronal vulnerability. Depending on dose and route (intraperitoneal vs. intracerebral), LPS induces acute or chronic neuroinflammation, dopaminergic sensitization or hippocampal-dependent cognitive deficits—phenotypes relevant to PD and AD, respectively [191,192,193]. LPS models are particularly valuable for studying TLR4/NF-κB signaling, complement activation, astrocytic reactivity, and glial–neuronal crosstalk, all of which represent therapeutic targets explored in clinical AD trials [103,194]. Other inflammatory stimuli, such as poly(I:C) (viral mimetic), CpG DNA, and NLRP3 inflammasome activators, produce distinct immune signatures that mimic viral, bacterial, or sterile inflammatory states (Figure 3). These models provide high construct validity for exploring neuroimmune pathways that contribute to sporadic AD, PD, and ALS and complement genetic models (e.g., TREM2, APOE, C9orf72) [195,196].

#### 3.3.2. Aging and Immunosenescence Models

Because aging is the strongest risk factor for all major neurodegenerative diseases, mouse strains that exhibit accelerated senescence—such as SAMP8 and SAMP10—have become valuable tools for studying age-related cognitive decline, synaptic dysfunction and low-grade neuroinflammation [197,198]. These mice develop oxidative stress, mitochondrial deficits, impaired proteostasis, and tau hyperphosphorylation. Models with defects in DNA repair or telomere maintenance (e.g., ERCC1, XPF, TERC−/−) reproduce hallmarks of human biological aging, including genomic instability, inflammaging, metabolic decline and vascular fragility. These features increase vulnerability to Aβ accumulation, tauopathy, motor neuron stress, or α-synuclein aggregation, depending on the background strain [199].

#### 3.3.3. Vascular and Neurovascular Unit Models

Cerebrovascular dysfunction is increasingly recognized as a major contributor to cognitive decline in AD and vascular dementia. In vivo systems that induce chronic cerebral hypoperfusion—such as bilateral carotid artery stenosis (BCAS) or two-vessel occlusion (2VO)—produce white matter damage, hippocampal dysfunction, BBB leakage, and glymphatic impairment [200,201]. These phenotypes mirror early vascular abnormalities observed in human AD and in APOE4 carriers. Endothelial-specific genetic models, pericyte-deficient mice, and BBB-breakdown models allow investigation of the neurovascular unit and its role in neurodegeneration, aligning closely with clinical findings on impaired Aβ clearance, tau propagation, and metabolic stress [187,188].

#### 3.3.4. Metabolic and Systemic Stress Models

Given strong epidemiological links between metabolic disorders and neurodegeneration, models incorporating high-fat diets, insulin resistance, mitochondrial toxins, or energy-restriction paradigms provide important translational relevance. Chronic metabolic stress induces synaptic vulnerability, impaired autophagy, mitochondrial damage, and glial activation—mechanisms synergizing with amyloid, tau, TDP-43, and α-synuclein pathologies. These models are valuable for studying the brain–liver axis, systemic inflammation, lipid metabolism, and peripheral immune modulation—key contributors to metabolic vulnerability in AD [202,203,204].

#### 3.3.5. Gut–Brain Axis Models

Animal models manipulating the microbiome—through germ-free conditions, antibiotic depletion, or fecal microbiota transplantation—have revealed profound contributions of microbial metabolites and immune signals to neurodegeneration. These paradigms modulate microglial maturation, amyloid plaque deposition, α-synuclein propagation along vagal pathways, and neuroinflammatory priming. Gut–brain axis models align with human findings linking dysbiosis, microbial amyloids, short-chain fatty acids, and systemic inflammation to AD, PD, and ALS progression [205,206].

#### 3.3.6. Multimodal and Combinatorial Models

To address limitations of single-mechanism systems, combined models integrate multiple pathogenic factors, such as

Amyloid + tau,

Amyloid + vascular,

Genetic mutation + inflammation,

α-synuclein + metabolic stress,

C9orf72 + immune activation.

These multi-hit paradigms better replicate nonlinear interactions in human neurodegenerative diseases. For example, combining *APOE4* expression with Aβ or tau pathology accelerates neurodegeneration and recapitulates human-like cognitive decline, vascular dysfunction, and neuroimmune activation [105,175]. Similarly, integrating neurotoxins with genetic susceptibility (e.g., MPTP + LRRK2) improves construct validity in PD [207]. Such models offer some of the highest translational value by approximating the multifactorial nature of disease progression observed in humans [208].

### 3.4. Translational Relevance and Integrative Perspective: Bridging Preclinical In Vivo Models with Alzheimer’s Disease Clinical Research

An integrative overview of key pathogenic mechanisms captured across toxin-based, genetic, and multimodal in vivo models—including mitochondrial dysfunction, protein aggregation, RNA dysmetabolism, neuroinflammation, and synaptic failure—is provided in Table 6.

A central challenge in neurodegeneration research is bridging mechanistic insights from preclinical models with therapeutic outcomes in human clinical trials. The combined framework of toxin-based, genetic, inflammatory, aging-related, vascular, metabolic, microbiome-based, and multitarget models creates a translational ecosystem that reflects key dimensions of human biology: synaptic resilience, neuroimmune regulation, proteostasis, mitochondrial function, cerebrovascular integrity, and systems-level metabolic signaling [209,210,211]. This integrative approach is particularly relevant to Alzheimer’s disease, which forms the focus of the subsequent clinical chapter (Section 4). Many pathogenic cascades modeled in vivo—Aβ accumulation, tau hyperphosphorylation, synaptic dysfunction, neuroinflammation, lipid dysregulation, BBB impairment, glymphatic flow reduction and aging-related vulnerability—correspond directly to clinical biomarkers used to define AD, including CSF Aβ42, p-tau, neurofilament light chain, GFAP, TSPO-PET, and vascular MRI signatures [212,213,214]. Preclinical in vivo systems also clarify why certain therapeutic strategies succeed or fail in human trials. For instance, amyloid-centric models reproduce plaque formation but often lack tau pathology, vascular dysfunction and metabolic decline → partially explaining limited efficacy of late-stage anti-Aβ therapies [215,216], while models incorporating inflammation, tau, lipid metabolism or mitochondrial impairment align more closely with integrated biomarkers observed in clinical AD cohorts [217,218].

Multimodal models that integrate amyloid, tau, neuroinflammation, vascular compromise, and aging effects provide the closest approximation to the multifactorial nature of human AD. Their value is especially evident in the interpretation of clinical trial responses to anti-amyloid antibodies (e.g., lecanemab, donanemab), where the interplay between amyloid clearance, downstream tau pathology, and neuroinflammation determines therapeutic impact [219,220,221]. Similarly, genetic models such as APOE4 knock-in mice mirror lipid dysregulation, BBB breakdown, microglial phenotypes, and metabolic vulnerability seen in humans, making them invaluable for genotype-stratified therapeutic evaluation and precision-medicine strategies in AD [175,222]. As clinical research advances toward multimodal interventions that combine anti-amyloid therapies with agents targeting tau, glia, mitochondria, vasculature, or metabolism, preclinical models provide the mechanistic basis for designing combination therapies and predicting interactions among therapeutic pathways [223,224].

Thus, transitioning from the in vivo systems described in this chapter to human clinical studies in AD (Chapter 4) is not a conceptual division but a mechanistic continuum. The mechanistic domains captured across in vivo models—summarized in Table 6—define the biological foundation for interpreting biomarker changes, understanding therapeutic variability, and designing next-generation disease-modifying treatments tailored to the complexity of AD [105,225,226,227,228,229].

**Table 6:** Mechanistic pathways modeled across in vivo neurodegeneration systems and their relevance to human disease.

Mechanistic Domain	Modeled in Toxin Systems	Modeled in Genetic Systems	Modeled in Integrative Systems	Relevance to Human Disease	Ref.
**Mitochondrial dysfunction**	MPTP, 6-OHDA, rotenone	PINK1, Parkin, SOD1	Aging models, metabolic stress	Central mechanism in PD, ALS, AD	[131,132,147,167,168,170,197,203,204]
**Oxidative stress**	Paraquat, 6-OHDA	SOD1, α-syn, APP	Inflammation, vascular injury	Universal neurodegeneration hallmark	[133,141,148,149,150,152,179]
**Protein aggregation**	α-syn induction	α-syn, APP/PSEN, tau, TDP-43, FUS, C9ORF72	Multi-hit amyloid + tau models	Core pathology in AD, PD, ALS, FTD	[155,156,157,158,160,161,209,212]
**Impaired proteostasis**	Proteasome inhibition	TDP-43, FUS SOD1, tau	Aging, metabolic dysfunction	Drives accumulation of misfolded protein	[155,156,157,179,182,189,190]
**RNA dysmetabolism and nucleocytoplasmic transport defects**	-	TDP-43, FUS, C9orf72	Aging, chronic inflammation	Core mechanism in ALS/FTD spectrum disorders	[98,180,181,182,183,184,185,186]
**Neuroinflammation**	LPS, poly(I:C)	APOE4, TREM2, C9ORF72, TDP-43, FUS	Gut–brain axis, vascular models	Major driver of progression across diseases	[103,193,194,195,217,226,227]
**Synaptic failure and neuronal loss**	Glutamate dysregulation	APP/PS1, tau, α-syn models	Hypoperfusion, metabolic stress, chronic inflammation	Strong predictor of cognitive decline, behavioral symptoms and motor dysfunction	[103,118,179,194,214,217,225]
**Cerebrovascular dysfunction**	Indirect (mitochondrial/oxidative injury)	APOE4	BCAS, 2VO, BBB disruption models	Early AD biomarker and contributor to cognitive decline	[187,188,201,211,215,216]
**Network degeneration**	Dopaminergic loss (MTPT, 6-OHDA)	Tauopathy models, α-syn propagation	Combined models	Mechanistic basis of clinical symptoms	[118,120,152,189,190,208,213]

## 4. Current and Investigated Neuroprotective Therapies in Neurodegenerative Diseases

The development of neuroprotective therapies is based on insights gained through experimental models described above, both in vitro and in vivo. In vitro assays have allowed the identification of mechanisms that are easy to work with and that can be made into drugs. Cellular systems are essential for identifying mechanisms such as oxidative stress, mitochondrial dysfunction, and aberrant protein aggregation. Meanwhile, animal models confirm the translational relevance of candidate compounds by assessing pharmacokinetics, behavioral outcomes, and BBB penetration. The development of therapeutics for neurodegenerative diseases has been driven by an iterative pipeline. In vitro systems (e.g., neuronal cell lines and primary cultures) support the discovery of mechanisms and the initial screening of compounds, while in vivo models establish exposure–response relationships, behavioral benefits, and safety prior to first-in-human trials [171,230]. For instance, cholinesterase inhibitors and N-methyl-D-aspartate (NMDA) antagonists were optimized using hippocampal/cortical preparations and excitotoxicity assays prior to translation. In parallel, human iPSC-derived neurons and brain organoids improve human-specific fidelity for target engagement and off-target liability assessment, and are increasingly used to triage candidates—including tau-directed modalities and targeted protein degraders—prior to animal and clinical studies [231,232,233]. Meanwhile, anti-amyloid monoclonal antibodies were prioritized based on plaque reduction and cognitive effects in amyloid precursor protein APP/PSEN knock-in or transgenic mice [171]. These then progressed to biomarker-anchored phase 3 programs [219]. Despite this complementary framework and modest rate of success, however, the translational efficiency of preclinical findings remains critically low: many compounds that demonstrate neuroprotective activity in cells or rodents fail to progress beyond the initial stages of clinical trials due to toxicity or insufficient efficacy in humans [234,235,236,237]. These challenges highlight the limitations of existing model systems and emphasize the need for model-informed, mechanism-based drug discovery. An alternative, increasingly pragmatic strategy is to repurpose clinically established drugs whose pharmacological and safety profiles are well understood. Screening such compounds for additional neuroprotective or anti-inflammatory activity, either on their own or in combination with symptomatic treatments, offers a way to speed up development and improve therapeutic outcomes.

While conventional therapeutic interventions mainly address symptoms, there has been a growing emphasis on developing DMTs that aim to inhibit neuronal degeneration or promote regeneration [238,239]. In this section, we summarize approved and investigational agents, particularly those evaluated in preclinical models, and discuss how evidence obtained from in vitro and in vivo systems informs their clinical translation. Only compounds studied for AD will be further discussed, as it is the most common neurodegenerative disease, with more than 55 million cases in 2024 [22]. An updated reviews of repurposing agents for PD [240] and other less frequent NDDs [241] have been recently published elsewhere. As the literature search engine, the ClinicalTrials.gov database was searched for currently ongoing and completed clinical trials (accessed 30 November 2025) [242].

### 4.1. Approved Therapeutic Agents in AD

**Figure 4:**
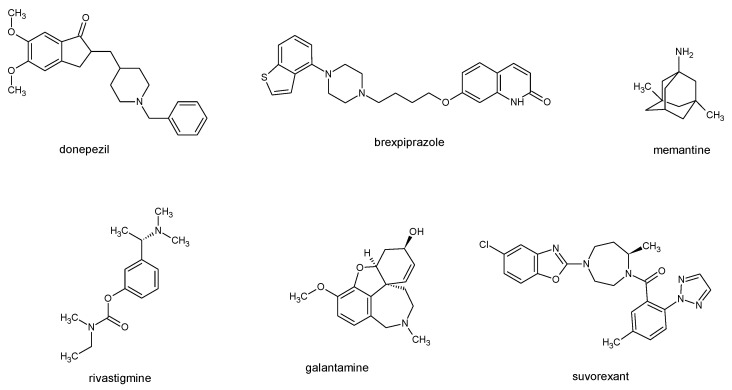
Chemical structure of the FDA-approved symptomatic drugs in AD. Created in ChemSketch^®^ v. 2025.

In the light of current theory of acetylcholine (ACh) deficits in the course of AD as a result of cholinergic neurons loss, most of the currently used symptomatic agents are cholinomimetics, which may enhance the neuronal signal mediated by ACh. In this group, galantamine, rivastigmine, and donepezil belong to the subclass of acetylcholinesterase inhibitors (AChEIs) and are of particular importance [243]. Galantamine is a naturally occurring plant secondary metabolite, derived from common snowdrop *Galanthus nivalis* and belongs to the alkaloid class [244]. It has unique, dual mechanism of action, because it is not only a selective and reversible AChEI but also an allosteric modulator of nicotinic receptor [245]. Such intricate influence promotes the release of additional neurotransmitters such as glutamate, dopamine, and serotonin, further improving neuronal communication and synaptic plasticity [246]. Donepezil is a second-generation AChEI that reversibly inhibits AChE [247]. It is approved for use across all stages of AD from mild to moderate and severe, showing a favorable safety profile, with mostly mild and transient gastrointestinal and neurological side effects, including nausea, dizziness, and headache [248]. Until recently there was only oral donepezil available; however, in 2022, the Food and Drug Administration (FDA) approved first donepezil transdermal patch, applied once weekly, which may assure better adherence and less frequent gastrointestinal side effects [249]. Rivastigmine can in turn inhibit both acetylcholinesterase and butyrylcholinesterase (BuChE). AChE and BuChE are distinctly expressed in certain regions of the central nervous system. AChE is mainly found in the neurons of the cerebral cortex; thus, BuChE is more predominantly located in glial cells [250]. This results in the potential for the treatment of other types of dementia, primarily in the course of PD [251]. Rivastigmine is also available in both oral and transdermal formulations; moreover, the rate of hepatic metabolism is negligible, which may be preferable in patients with polypharmacy and high risk of drug–drug interactions [252]. Although side effects of these medications are mostly transient and dose-dependent, certain features as hallucinations or dysphagia after donepezil or rivastigmine administration may be persistent and leads to discontinuation; therefore, novel dosage forms (e.g., nasal spray) are proposed as better tolerated alternatives [253]. On the other hand, neurotoxicity in AD and related cognitive impairment is also caused by overactivation of glutamate receptors, mainly NMDA, which leads to calcium and sodium overload in neurons, ultimately resulting in excitotoxicity and cell death [254]. Memantine is an uncompetitive antagonist of NMDA receptors and is widely used in all stages of AD, slowing the decrease in cognitive performance and memory loss [255,256]. Memantine is also well-tolerated, available in oral formulation, and can be combined with AChE inhibitors to improve its efficacy in alleviating AD symptoms [257]. To the FDA-approved drugs in AD also belong the atypical antipsychotic agent brexpiprazole and orexin receptor antagonist suvorexant for alleviating agitation and insomnia related to AD, respectively [258]. The chemical structures of currently small-molecule FDA-approved agents in AD are shown in Figure 4.

Apart from delaying AD-related dementia and cognitive impairment, DMTs have been proposed. To date, three humanized monoclonal IgG1 antibodies targeting Aβ have been approved by the FDA—aducanumab, lecanemab, and, most recently, donanemab [259]—with certain distinctions in molecular targets, dosing regimen, indications, and side effects. Aducanumab was the first-in-class DMT agent in AD, targeting insoluble Aβ aggregates and plaques [260,261]; however, controversies arose regarding its approval procedure due to insufficient safety and efficacy data, so it has been withdrawn from market in early 2024 [262,263,264]. Lecanemab, in turn, targets soluble Aβ protofibrils, which are perceived as more toxic than insoluble aggregates or monomers, and it was approved by the FDA in 2023 [265,266]. In July 2024, the third-in-class donanemab was authorized by the FDA as an anti Aβ agent [267] after consideration based on the results of TRAILBLAZER-ALZ 2 phase III randomized clinical trial (RCT) [221]. Donanemab targets pyroglutamyl(3)-Aβ (pGlu3-Aβ) protein (3–42) in order to reduce and fully remove Aβ plaques. This mechanism of action allows for the discontinuation of treatment when the Aβ deposits are cleared [268], which could be a distinct advantage compared to other molecules in class. While they have shown the ability to reduce amyloid plaques and modestly slow cognitive decline [269], they are limited by safety concerns like amyloid-related imaging abnormalities (ARIA), high treatment costs, as well as the need for early diagnosis and regular monitoring [270,271]. Their benefits are primarily observed in patients with mild cognitive impairment or early dementia; however, the reversion of the disease is not observed [259]. Taking into account serious concerns regarding efficacy and safety of anti-Aβ antibodies, further evaluation of this class is needed to undoubtedly confirm the rationale for their utilization. In addition, in China, an oral oligosaccharide derived from marine algae, sodium oligomannate (GV-971), was approved in 2019 for the treatment of mild to moderate AD [272]. It modulates the gut–brain axis by remodeling the gut microbiota and regulating amino acid metabolism. This reduces peripheral levels of phenylalanine and isoleucine, which are elevated in AD, and stimulates the proliferation of pro-inflammatory Th1 cells. Th1 cells in turn activate M1-type microglia and contribute to neuroinflammation, which is a key driver of AD pathophysiology. In the mice models of AD, oral administration of 50–100 mg/kg of body weight daily significantly reduced both peripheral and central nervous system (CNS) levels of proinflammatory cytokines as well as promoted abundance of certain bacteria, for instance Bifidobacterium spp. GV-971. It also inhibits amyloid-β fibril formation and promotes the clearance of Aβ aggregates, thereby offering anti-inflammatory and disease-modifying effects [273,274,275]. The results of a phase III RCT (NCT0229391) demonstrated the efficacy of GV-971 in improving cognitive function in patients with AD; however, AD biomarkers were not assessed in this study [276], and, to our knowledge, it is currently approved only in China [277].

### 4.2. Repurposing and Repositioning of Existing Drugs

Drug repurposing, also commonly known as drug repositioning, refers to the strategy of identifying new therapeutic uses for drugs that have already been discovered. Since 2004, repurposing has gained significant attention after Ashburn and Thor provided comprehensive work outlining the benefits and opportunities of investigating known molecules for uses other than those originally intended [278]. However, although the terms ‘repurposing’ and ‘repositioning’ are often used interchangeably, repurposing refers to drugs that have already been approved and marketed, while repositioning also covers drug candidates that have been discontinued or failed in their original applications [279]. As repositioning is considered the broader and more common definition in scientific literature [280], we will use this nomenclature in the subsequent section.

In this section, we summarize the main drug classes that have been repurposed to target key pathological processes in AD. Only compounds that have entered any phase of clinical trials will be discussed. Antidiabetic drugs (e.g., metformin, thiazolidinediones, SGLT2 inhibitors, and GLP-1 receptor agonists) are primarily proposed to restore energy homeostasis and insulin signaling, thereby mitigating downstream oxidative stress, neuroinflammation, and synaptic dysfunction. Anti-inflammatory and immunomodulatory agents (e.g., non-steroidal anti-inflammatory drugs (NSAIDs), cromolyn/ibuprofen combinations, Janus kinase (JAK) inhibitors, tumor necrosis factor (TNF)-α inhibitors, phosphodiesterase (PDE) 4 inhibitors, and leukotriene antagonists) aim to reduce microglial activation and cytokine-driven neurotoxicity, which contribute to neuronal injury and disease progression. Tyrosine kinase inhibitors and senolytic strategies (e.g., nilotinib, masitinib, and dasatinib plus quercetin) are being investigated for their potential to enhance proteostasis and autophagic clearance, as well as modulate inflammatory signaling. Finally, neuropsychiatric and network-stabilizing drugs (e.g., levetiracetam, bromocriptine, and atomoxetine) target circuit hyperexcitability and neurotransmitter dysregulation—mechanisms that are increasingly recognized in early AD. However, clinical translation across these classes has been limited by factors such as insufficient brain exposure, patient heterogeneity, and endpoint sensitivity.

Table 7 provides a synoptic overview of repurposed drug classes and their translational challenges, while Supplementary Table S1 presents trial-level details.

### 4.3. Novel DMTs in AD

DMTs are the most promising approach in the search for effective AD treatment. Rather than merely alleviating symptoms, they aim to alter the underlying pathology and progression of the disease. According to the 2024 Alzheimer’s Drug Development Pipeline Report, 141 DMTs are currently under investigation in various phases of clinical trials [297]. These therapies span a broad range of mechanisms, including targeting amyloid and tau, modulating neuroinflammation, supporting synapses and neurotrophins, and regulating metabolism. This diversity in DMT candidates reflects our evolving understanding of AD as a multifactorial disorder requiring multimodal therapeutic approaches. As reviewing drugs currently being investigated in any clinical or preclinical phase is beyond the scope of this paper, we will only briefly summarize compounds in the most advanced stages of development and one of the most promising approaches in novel research strategies, such as PROTACs. Readers are referred elsewhere for comprehensive information [230,297,298].

The development of anti-amyloid therapies continues to be prioritized as a key area in AD drug research, with the aim of reducing the Aβ burden and its neurotoxic effects [299]. Monoclonal antibodies such as remternetug (LY3372993), gantenerumab, and crenezumab target aggregated Aβ in order to promote plaque clearance [300,301,302]. Small molecules such as CT1812 act by antagonizing the sigma-2 receptor to displace Aβ oligomers from synaptic receptors [303]. Meanwhile, active immunotherapies such as UB-311 aim to stimulate an adaptive immune response [304]. Other investigational agents, including ALZ-801 and ACU193, focus on inhibiting oligomers selectively [305,306]. Anti-tau therapies are becoming increasingly important in the development of drugs for AD, targeting the aggregation and spread of the pathological tau protein, which is closely correlated with cognitive decline. Monoclonal antibodies such as E2814, semorinemab, zagotenemab, and JNJ-63733657 are designed to neutralize extracellular tau protein and prevent its propagation across synapses [307,308,309,310]. Other strategies include small molecules such as LM11A-31, which aim to inhibit tau aggregation or modulate tau phosphorylation [311]. Therapies that target neuroinflammation address the chronic activation of microglia and astrocytes, which contributes to neuronal damage in AD. Investigational agents such as NE3107, a small molecule with combined anti-inflammatory and insulin-sensitizing activity, aim to modulate proinflammatory signaling pathways [312]. Other compounds, such as AL002, a TREM2 agonist antibody, target microglial function in order to enhance debris clearance and restore immune homeostasis [313]. Other approaches include P2X7 and P2Y receptor modulators, as well as inflammasome inhibitors, which all aim to reduce harmful neuroimmune responses. However, they have not reached clinical evaluation yet [314,315,316,317]. Another DMT strategy targets neuroplasticity, which encompasses synaptic remodeling, dendritic spine dynamics, and the functional reorganization of neural circuits [318]. It is a fundamental process that is disrupted early in AD and is now one of the key focuses in therapeutic development, as loss of synaptic integrity strongly correlates with cognitive decline and often precedes neuronal death. This makes preserving and restoring neuroplastic mechanisms a compelling intervention strategy [319]. Several investigational agents aim to directly modulate pathways involved in synaptic maintenance and plasticity. For example, dalzanemdor (formerly SAGE-718) is an NMDA receptor positive allosteric modulator designed to enhance excitatory neurotransmission and support synaptic efficacy [320] also studied in Hutington’s disease [321]. Meanwhile, ACD856 targets tropomyosin receptor kinase B (TrkB) signaling downstream of BDNF, which is a critical mediator of activity-dependent synaptic strengthening and neuronal survival [322].

Proteolysis-targeting chimeras (PROTACs) are a novel therapeutic strategy for AD, enabling the selective degradation of pathological proteins involved in neurodegeneration. Unlike traditional inhibitors, PROTACs in the cell (e.g., neurons) utilize the ubiquitin–proteasome system to identify and eliminate specific targets, providing greater potency and fewer off-target effects [323]. In AD, the tau protein is a key target for PROTAC-based degradation, and compounds such as QC-01-175 or C8 have demonstrated the ability to selectively degrade tau, thereby reducing its aggregation and toxicity [324,325]. This approach reduces the pathological burden and may mitigate downstream neuroinflammation and synaptic dysfunction. However, despite their promise, PROTACs face several challenges in AD therapy. These include poor blood–brain barrier penetration due to their large molecular size and limited neuronal expression of essential E3 ligases. Furthermore, concerns remain regarding off-target effects, metabolic instability, and the potential long-term consequences of protein degradation in the brain [297]. When it comes to CNS translation, there are three main issues to consider. Firstly, BBB delivery and unbound brain exposure are often limiting factors because PROTACs are typically large and polar molecules that may be subject to efflux. Therefore, brain pharmacokinetics (PK) must be engineered and verified directly (i.e., brain/CSF exposure and brain target engagement) rather than inferred from plasma PK [326,327]. Secondly, efficacy depends on the availability of CNS-relevant E3 ligases (i.e., cell-type/region expression and intracellular compatibility), and the choice of ligase can also influence off-target degradation. This makes ligase selection and proteome-wide off-target checks a core step early in development. Thirdly, the PK/PD relationship is more complex for degraders due to exposure-dependent ternary complex formation with the potential for a ‘hook effect’, so time-resolved degradation readouts should accompany exposure measurements to inform dosing [325,327,328]. In practice, delivery solutions follow a stepwise approach: first, CNS-oriented medicinal chemistry is employed to minimize polarity and H-bonding, as well as optimize linker architecture. Then, prodrug/masked-polarity or BBB-delivery approaches are considered (e.g., shuttle- or carrier-based strategies), when chemistry alone cannot achieve adequate brain exposure [326].

**Figure 5:**
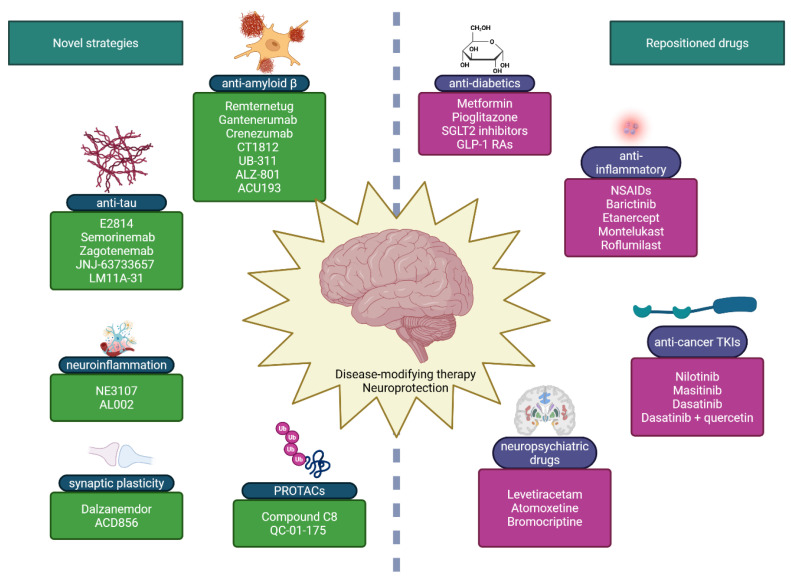
An overview of the current clinical investigations into DMTs in AD. Created in BioRender^®^.

These limitations highlight the importance of careful optimization and targeted delivery strategies for CNS applications. To date, no PROTAC has reached the stage of clinical trials, and only data from preclinical studies is available; however, it is one of the most important future strategies for DMT development in AD [328]. A schematic summary of current investigational strategies, including both repositioning and novel compounds development DMT strategies, is shown in Figure 5.

## 5. Conclusions and Future Perspectives

Despite ongoing research, the clinical success rate of neuroprotective drug candidates remains disappointingly low. Repeated late-stage failures highlight the limited predictive validity of current preclinical models, which frequently only capture narrow aspects of human neurodegeneration and neglect comorbidities, species-specific pharmacokinetics, and patient heterogeneity [230,298]. Therapeutic development is characterized by high attrition across major neurodegenerative diseases, especially when moving from mechanistic promise to clinically meaningful endpoints. In AD, an analysis of trials registered with ClinicalTrials.gov from 2002 to 2012 reported an overall success rate of 0.4% (99.6% failure) across phases [329]. Similarly, a subsequent analysis of phase II/III AD dementia programs (2004–2021) identified 98 failed compounds and estimated an approximate 2% success rate in phases II/III since 2003 (two ‘successes’ versus 98 failures) [330]. By contrast, programs for PD show relatively higher progression and approval rates, yet substantial attrition remains. During the 1999–2019 period, 152 compounds in 357 trials yielded an overall success rate of 14.9% (i.e., ~85% did not reach approval), with repurposed compounds reaching approval less frequently than original compounds (6.7% vs. 21.4%) [331]. On the other hand, SH-SY5Y neuronal models, particularly those differentiated with RA/BDNF, are used for the rapid, mechanistic screening of repurposed candidates under Aβ stress and then advanced to amyloid mouse models. This staged approach has directly supported repurposing programs such as those involving GLP-1RAs [332,333] and metformin [334]. Human-relevant systems, such as iPSC-derived neurons, 3D co-cultures, and brain organoids, offer greater physiological relevance by replicating human cell types and network interactions. However, they still lack full maturation, vasculature, and immune components [231,233]. Integrating multi-omics analyses with machine learning further enables cross-validation between model- and patient-derived datasets, improving translational alignment [335].

In clinical practice, biomarker-driven enrichment and adaptive trial designs based on the AT(N)/(I) framework, cerebrospinal and plasma markers, and imaging endpoints can substantially enhance trial sensitivity [336,337]. In parallel, digital biomarkers, including the continuous monitoring of cognitive and motor parameters, have emerged as powerful tools for bridging the gap between laboratory endpoints and real-world trajectories [338,339]. This translational roadmap focuses on targeted protein degradation, particularly through PROTACs, as an innovative and promising therapeutic approach [340]. PROTACs recruit endogenous E3 ubiquitin ligases to pathogenic proteins, resulting in the selective degradation of these proteins rather than their inhibition. This strategy enables the modulation of previously ‘undruggable’ targets, such as tau or TDP-43 aggregates, which are implicated in neurodegenerative diseases [326,341]. Proof-of-concept studies demonstrate that brain-directed PROTACs can reduce protein aggregation and neurotoxicity in vitro and in vivo, suggesting genuine disease-modifying potential [342]. However, there are still some critical challenges to overcome in the translation of this approach into clinical practice: ensuring effective BBB penetration and optimal pharmacokinetic profiles for large degrader molecules [327]; identifying CNS-compatible E3 ligases to minimize off-target degradation [231,343]; and demonstrating functional benefit beyond target clearance in models that replicate human disease progression [326]. Furthermore, the multifactorial and complex nature of neurodegeneration, which spans aging, inflammation, and metabolic dysregulation, necessitates the integration of PROTAC strategies within precision-medicine frameworks anchored in biomarkers. The current evidence suggests that the convergence of human-relevant models, multi-omics integration, adaptive clinical design, and novel modalities such as PROTACs may finally bridge the translational gap between laboratory findings and effective neuroprotective therapies.

## Figures and Tables

**Table 2:** Comparative evaluation of in vitro neurodegeneration models based on structural, genetic, and functional attributes.

Feature/ Model	Immortalized Cell Lines	Primary Neurons	iPSC-Derived Neurons	Co-Cultures	Three-dimensional Spheroids	Microfluidic Chips	Organoids	Ref.
**Species**	Human/rodent (tumor origin)	Rodent	Human (patient-specific)	Human/ rodent	Human/rodent	Human/ rodent	Human	[27,37,40,46,47,53,54,88,89,112]
**Neuronal maturity**	Low–moderate	High	Variable (improves over time)	Moderate–high	Moderate–high	Variable	Moderate (increases with long-term culture)	[30,46,48,53,54,88,89,90,112]
**Genetic relevance**	Limited	Rodent-inherent	High	High	High	High	Very high	[23,39,40,44,45,53,54,99]
**Multicellularity**	None	Partial (glia present naturally)	Optional (astrocytes, microglia)	Yes	Emerging	Controlled (compartmentalized)	High	[57,58,59,60,62,63,64,67,93]
**Three-dimensional architecture**	No	No	No	No	Yes	Partial	Yes	[71,72,73,88,89,90,92,95,104]
**Modeling protein aggregation**	Limited	Better	High	High	High	High	Very high	[23,34,42,95,99,100,104,105,106,107]
**Modeling neuroinflammation**	Poor	Moderate	High (with microglia)	High	Moderate	High (neuron–glia communication)	High (with microglia-containing organoids)	[62,63,64,65,66,67,68,93,113]
**Throughput**	Very high	Low	Low–medium	Medium	Medium	Low–medium	Low	[25,27,41,53,54,112]
**Technical difficulty**	Low	High	Very high	Medium	Medium	High	Very high	[46,47,48,53,77,88,89]
**Cost**	Low	Low–medium	High	Medium	Medium	Medium-high	High	[25,40,41,53,88,89]

**Table 5:** Genetic in vivo models of neurodegeneration and their translational relevance.

Gene/Pathway	Model Type	Key Phenotypes	Relevance to Human Disease	Ref.
***α-Synuclein*** **(A53T, A30P, E46K)**	Transgenic mice; viral vector overexpression	Aggregation, motor deficits, synaptic dysfunction	Lewy body pathology; prion-like propagation	[158,159,160]
***LRRK2* (G2019S, R1441C/G)**	Knock-in; overexpression	Altered dopamine release, axonal defects	Familial PD; kinase-targeted therapy testing	[162,163,164,166]
***Parkin/PINK1***	Knockout/knockdown	Mitophagy failure, synaptic changes	Early PD mechanisms; mitochondrial quality control	[61,167,168]
***APP/PSEN1/PSEN2***	Transgenic lines (APP/PS1, 5xFAD, Tg2576)	Aβ plaques, gliosis, cognitive decline	Familial AD mechanisms; amyloid-targeted therapies	[171,172,173]
***APOE4***	Humanized knock-in	BBB leakage, neuroinflammation, lipid dysregulation	Sporadic AD risk; genotype-stratified trials	[174,175,187,188]
***Tau* (P301S, P301L)**	Overexpression	NFT formation, neuron loss	Tauopathy; anti-tau drug development	[161,176,177]
***SOD1^G93A***	Transgenic overexpression	Motor neuron death, paralysis	Oxidative stress and proteostasis in ALS	[120,152,179]
***TDP-43***	Overexpression/ knock-in	Aggregation, synaptic failure	ALS/FTD proteinopathy	[180,181,182,183]
***FUS***	Transgenic overexpression; knock-in	RNA dysmetabolism; impaired DNA damage response; stress granule pathology; synaptic dysfunction; motor and cognitive deficits	ALS/FTD proteinopathy; nucleocytoplasmic transport defects; RNA-binding protein toxicity	[180,181,182,183]
***C9ORF72***	Repeat expansion; knockdown	RNA foci, DPRs, behavioral deficits	Most common ALS/FTD mutation	[184,185,186]

**Table 7:** An overview of drug classes being repositioned in AD.

Drug Class	Representative Drugs	Primary Mechanism	Most Suitable Experimental Models	CT Phase/Endpoint	Main Translational Limitation	Ref
**Biguanides**	Metformin	AMPK activation; improved insulin signaling; reduced neuroinflammation	iPSC-derived neurons; mixed neuronal–glial cultures; metabolic stress models	Phase II; cognitive and executive function outcomes	Small/heterogeneous cohorts and modest signals; unclear CNS target engagement	[281,282]
**Thiazolidinediones**	Pioglitazone	PPARγ activation; anti-inflammatory and mitochondrial effects	AD transgenic mice; astrocyte–microglia co-cultures	Phase III (TOMMORROW); MCI prevention	Prevention trials require long follow-up and high-risk enrichment; metabolic confounding; limited signal in AD	[283,284]
**GLP-1 RAs**	Liraglutide; semaglutide	GLP-1R activation; synaptic protection; reduced neuroinflammation; metabolic reprogramming	iPSC cortical neurons; AD mouse models	Phase IIb–III; CDR-SB, FDG-PET	BBB exposure/target engagement in brain variable; clinical endpoints may require longer duration and biomarker stratification	[285,286]
**SGLT2 inhibitors**	Dapagliflozin; empagliflozin	Systemic metabolic shift; reduced oxidative stress; vascular protection	iPSC neurons; early AD biomarker models	Phase II; neuroimaging and cognitive endpoints	Delivery method/absorption variability; insulin resistance heterogeneity; risk of unblinding with nasal effects; short trial duration	[287,288]
**Anti-inflammatory agents**	Montelukast; roflumilast, tarenflurbil	Leukotriene receptor antagonism; PDE4 inhibition; microglial modulation; cyclooxygenase inhibition	Microglial cultures; LPS-induced neuroinflammation models	Phase II; cognitive outcomes	Mixed translation from inflammation-only models to AD; need biomarker-defined “inflammatory AD” and BBB exposure data, uncertain/insufficient CNS target engagement	[289,290]
**TKIs**	Nilotinib; masitinib	Autophagy induction; proteostasis and neuroinflammation modulation	SH-SY5Y cells; iPSC neurons; AD mouse models	Phase II–III; cognition and biomarker endpoints	BBB penetration/efflux and dose-limiting AEs; off-target effects	[291,292]
**CNS drugs**	Levetiracetam, atomoxetine, bromocriptine	Network stabilization and reduction in neuronal hyperexcitability; NET inhibition → ↑ CNS norepinephrine; D2 agonists	Hippocampal neuron cultures; EEG-informed AD models	Phase II; memory and EEG endpoints	Key limitation: effects may be subgroup-specific (hyperexcitability, LC dysfunction, PSEN1-AD) and require longer, powered parallel-arm trials.	[293,294,295]
**Senolytics**	Dasatinib + quercetin	Clearance of senescent cells; suppression of SASP	iPSC-derived aging models; aged mice	Phase I–II; biomarker endpoints	Early-phase safety/biomarker trials; small sample size; short duration	[296]

↑—increasing.

## Data Availability

No new data were created or analyzed in this study. Data sharing is not applicable to this article.

## Supplementary Materials

The following supporting information can be downloaded at https://www.mdpi.com/article/10.3390/molecules31020320/s1, Supplementary Table S1. Clinical trials of possible compounds to be repositioned as DMTs in AD. Refs. [281,282,283,285,286,287,289,291,294,295,344,345,346,347,348,349,350,351,352,353,354,355,356,357,358,359] are cited in Supplementary Materials.

## Abbreviations

6-OHDA	6-hydroxydopamine
ACh	Acetylcholine
AChEIs	Acetylcholinesterase inhibitors
AD	Alzheimer’s Disease
ADHD	Attention deficit/hyperactivity disorder
AF64A	Ethylcholine aziridinium
ALS	Amyotrophic Lateral Sclerosis
APP	Amyloid precursor protein
ARIA	Amyloid-related imaging abnormalities
Aβ	Beta amyloid
BBB	Blood–brain barrier
BDNF	Brain derived neurotrophic factor
BuChE	Butyrylcholinesterase
CMRglc	Cerebral glucose metabolism rate
CNS	Central nervous system
COX	Cyclooxygenase
CSF	Cerebrospinal fluid
CysLT1	Cysteinyl leukotriene receptor 1
DAT	Dopamine transporter
dbcAMP	Dibutyryl cyclic adenosine monophosphate
DPRs	Dipeptide repeat proteins
FAD	Familial Alzheimer’s disease
fALS	Familial amyotrophic lateral sclerosis
FDA	Food and Drug Administration
FTD	Frontotemporal Dementia
GDNF	Glial cell-derived neurotrophic factor
GLP-1	Glucagon-like peptide-1
GLP-1RAs	Glucagon-like peptide-1 receptor agonists
GRN	Progranulin
iNOS	Inducible nitric oxide synthase
JAK	Janus kinase
L-DOPA	L-3,4-dihydroxyphenylalanine
LEV	Levetiracetam
LRRK2	Leucine-rich repeat kinase 2
LUHMES	Lund Human Mesencephalic cells
MAO-B	Monoamine oxidase-B
MAPT	Microtubule-associated protein tau
MDA	Malondialdehyde
MPP+	1-methyl-4-phenylpyridinium
MPTP	1-methyl-4-phenyl-1,2,3,6-tetrahydropyridine
NDD	Neurodegenerative disorder
NGF	Nerve growth factor
NMDA	N-methyl-D-aspartate
NSAIDs	Non-steroidal anti-inflammatory drugs
PD	Parkinson’s disease
PDE4	Phosphodiesterase 4
PINK1	Pten-induced kinase 1
PPARγ	Peroxisome proliferator-activated receptor gamma
PRKN	Parkin
PROTACs	Proteolysis-targeting chimeras
PSEN1 and PSEN2	Presenilin genes
QE	Quercetin
RA	Retinoic acid
RCT	Randomized clinical trial
ROS	Reactive oxygen species
SAD	Sporadic Alzheimer’s disease
sALS	Sporadic amyotrophic lateral sclerosis
SGLT-2	Sodium-glucose co-transporter 2
SNCA	α-synuclein
SOD1	Superoxide dismutase 1
SV2A	Synaptic vesicle protein 2A
T2DM	Ttype 2 diabetes mellitus
TH	Thyrosine hydroxylase
TKIs	Tyrosine kinase inhibitors
TPA	12-O-tetradecanoyl phorbol acetate
TrkB	Tropomyosin receptor kinase B
TZDs	Thiazolidynediones
VMAT2	Vesicular monoamine transporter type 2
