# Supplementary material for: Experimental Models and Translational Strategies in Neuroprotective Drug Development with Emphasis on Alzheimer’s Disease
Source: Molecules. 2026 Jan 16;31(2):320. doi: 10.3390/molecules31020320 (PMC12844234; doi:10.3390/molecules31020320)
Supplement: Supplementary file 1 [file molecules-31-00320-s001.zip › molecules-4049094-supplementary.pdf]

molecules

MDPI

Supplementary Table S1. Clinical trials of possible compounds to be repositioned as DMTs in AD.

Drug	NCT ID/ study name	Phase	Interven-tion	Study dura-tion	Participants	Main outcomes	Ref.
**Metformin**	NCT00620191	Pilot trial	1000 mg BID	12 months	80 patients with amnestic MCI	No significant differ-ences in ADAS-Cog, rCMRglc or plasma Aβ42.	[281]
	NCT01965756	Pilot trial	Start from 500 mg QD increased to 1000 mg BID	8 weeks	20 subjects with MCI or early dementia without diabe-tes	**Improvement in executive function-ing**. CBF and CSF Aβ42, total tau and p-tau did not differ significantly.	[282]
**Rosiglita-zone**	REFLECT-1 NCT00428090	III	2 mg or 8 mg rosiglitazone XR QD	24 weeks	693 subjects divided by APOE-ε4 – positive and negative	**No significant changes** in ADAS-Cog and CIBIC+ cognitions scores	[344]
**Pioglitazone**	TOMMOR-ROW NCT01931566	III	0.8 mg pioglitazone SR QD	5 years	3494 partici-pants with high or low risk for devel-oping MCI due to AD	**No significant delay** onset of MCI versus placebo, **terminated preterm**	[283]
**Dapagli-flozin**	NCT03801642	I/II	10 mg QD	12 weeks	48 subjects with probable AD measured by MMSE score	**No changes** in cere-bral N-acetylaspartate level measured by MRI spectroscopy	[287]
**Liraglutide**	ELAD NCT01843075	IIb	1.8 mg QD s.c. vs place-bo	12 months	204 patients with mild AD without diabe-tes	The primary end-point (CMRglc) **did not improve.** There was a significant change in grey matter loss and an improvement in the ADAS-EXEC score. Only preliminary results are available.	[285, 345,346]

 https://doi.org/10.3390/molecules31020320

Drug	NCT ID/ study name	Phase	Interven-tion	Study dura-tion	Participants	Main outcomes	Ref.
**Semaglutide**	EVOKE & EVOKE+ NCT04777396, NCT04777409	III	14 mg QD oral semaglutide vs placebo	104 weeks	3808 patients with MCI	Large, pivotal trials of oral semaglutide in early AD, **results not posted yet**	[286]
**Tarenflurbil (R-flurbiprofen)**	NCT00322036	III	800 mg BID	18–24 months	1684 subjects with mild to moderate AD severity	Well tolerated**, no significant benefit on cognition** vs placebo	[347]
**Cromolyn + ibuprofen (ALZT-OP1)**	NCT02482324	I	Cromolyn 17–34 mg inhaled; ibuprofen 10–20 mg orally QD	2 days	26 healthy elderly volunteers	Good safety, CSF penetration of cromolyn and ibuprofen	[348]
	COGNITE NCT02547818	III	Cromolyn 17 mg inhaled; ibuprofen 10 mg orally QD	72 weeks	620 subjects with early AD	**No results posted yet**, trial has been completed in November 2020	[349]
**Roflumilast**	ROMEMA NCT04658654	II	50 µg QD or 100 µg QD vs placebo	24 weeks	81 patients with MCI	Ongoing	[350]
**Montelukast**	EMERALD NCT03991988	IIa	Oral 10 → 20 → 40 mg QD (titrated)	12 months	32 subjects with MCI or early AD	Ambiguous, **no significant changes** in CSF amyloid, tau or CDR score vs placebo, no peer reviewed results posted	[289]
**Montelukast (buccal film)**	BUENA NCT03402503	IIa	10 mg QD and 30 mg BID	26 weeks	70 patients with mild-to-moderate AD	No peer-reviewed results posted	[351]
**Baricitinib**	NADALS NCT05189106	II	2 mg QD for 8 weeks → 4 mg QD for 16 weeks	24 weeks	20 patients with MCI	No results posted yet	[352]

https://doi.org/10.3390/molecules31020320

Drug	NCT ID/ study name	Phase	Interven-tion	Study dura-tion	Participants	Main outcomes	Ref.
**Nilotinib**	NCT02947893	II	150 mg QD for 6 months → 300 mg QD for 6 months	12 months	37 subjects with mild-to-moderate AD	Well tolerated, measurable CSF nilotinib; **reduced** CSF Aβ40/42 and p-tau, reduced hippocampal atrophy; **no significant cognitive benefit** vs placebo	[353]
**Nilotinib BE (modified formulation)**	NILEAD NCT05143528	III	84 or 112 mg QD	72 weeks	1200 subjects with early AD	Large, ongoing trial to test disease-modifying effect with CDR-SB and amyloid/tau PET endpoints; **no results yet**	[291]
**Masitinib**	AB09004 NCT01872598	III	4.5 mg/kg QD orally	24 weeks	718 subjects with mild-to-moderate AD	Add-on to standard therapy; **significantly less decline** on ADAS-Cog and ADCS-ADL vs placebo; **AEs higher** but mostly manageable	[354]
**Dasatinib + quercetin**	SToMP-AD NCT04063124	I	Dasatinib 100 mg + quercetin 1250 mg PO once daily for 2 days every 2 weeks (6 cycles)	12 weeks	5 subjects with early AD	Dasatinib detected in CSF, **no powered cognitive outcome**	[355]
	STAMINA NCT05422885	I	As above	12 weeks	12 patients with MCI	D+Q feasible and safe; **preliminary improvement** in mobility and cognitive measures	[356]
	ALSENLITE NCT04785300	I	As above	12 weeks	15 patients with symptomatic AC	Ongoing, primary completion in June 2026	[357]

https://doi.org/10.3390/molecules31020320

Drug	NCT ID/ study name	Phase	Interven-tion	Study dura-tion	Participants	Main outcomes	Ref.
**Levetirace-tam**	LEV-AD NCT02002819	II	125 mg BID vs placebo	4 weeks	34 patients with mild AD	**Improvement** in spatial memory and executive function only in **prespecified subgroup**	[358]
	HOPE4MCI NCT03486938	IIb	220 mg QD (extended-release) vs placebo	78 weeks	164 subjects with MCI	Primary endpoint (CDR-SB**) not met overall**; post-hoc analyses suggest more favorable effect in APOE ε4 non-carriers	[359]
**Atomoxe-tine**	Atomoxetine in MCI NCT01522404	II	Flexible titra-tion 10–100 mg QD	6 months	39 patients with amnestic MCI	Well tolerated; ro-bust improvement in CSF norepinephrine and lowered CSF tau / p-tau, but **no sig-nificant cognitive improvement** vs placebo over 6 months	[295]
**Bromocrip-tine**	REBRAnD NCT04413344	I/II	Titration to 10–22.5 mg QD	52 weeks	12 patients with PSEN1 familial AD	Acceptable safety in PSEN1-AD	[294]

Abbreviations: ADAS-cog - Alzheimer's Disease Assessment Scale-cognitive subscale, rCMRglc - relative Cerebral Glucose Metabolism, MCI – mild cognitive impairment, CSF – cerebrospinal fluid, CBF – cerebral blood flow, XR – extended release, CIBIC+ - Clinician's Interview-Based Impression of Change plus caregiver input, MMSE - Mini-Mental State Examination, CDR Score – Clinical Dementia Rating Score, AEs – adverse events, ADCS-ADL - Alzheimer's Disease Cooperative Study Activities of Daily Living Inventory scale, D+Q – dasatinib+quercetin, ADAS-EXEC - ADAS-Cog with Executive domains of the Neuropsychological Test Battery, QD – once a day, BID – twice a day

https://doi.org/10.3390/molecules31020320
